# Predictors of Parental Barriers to Reduce Excessive Child Screen Time Among Parents of Under-Five Children in Selangor, Malaysia: Cross-sectional Study

**DOI:** 10.2196/25219

**Published:** 2021-04-13

**Authors:** Elliza Mansor, Norliza Ahmad, Diana Raj, Nor Afiah Mohd Zulkefli, Zalilah Mohd Shariff

**Affiliations:** 1 Department of Community Health Faculty of Medicine and Health Sciences Serdang Malaysia; 2 Ministry of Health Malaysia Putrajaya Malaysia; 3 Department of Nutrition and Dietetics Faculty of Medicine and Health Sciences Universiti Putra Malaysia Serdang Malaysia

**Keywords:** child, self-efficacy, screen time, Malaysia, parent-child relations, public sector, children, screen, parental

## Abstract

**Background:**

Globally, there is an increasing prevalence of excessive screen time exposure among young children, including in Malaysia. Parents are advised to limit this exposure, but there are barriers for many of them to follow this recommendation. To date, there is a lack of research on the factors that cause these parental barriers.

**Objective:**

This study aimed to determine the parental barrier toward the reduction of excessive child screen time and its predictors among parents of children aged younger than 5 years in the Petaling District, Selangor, Malaysia.

**Methods:**

A cross-sectional study was conducted from April 2019 to June 2020 among 789 parent-child dyads attending child health clinics in the Petaling District. Validated self-administered questionnaires were used to capture information on sociodemographic, parental, child-related, and environmental factors and parental barriers. Stratified sampling with probability proportionate to size was employed. Data were analyzed using SPSS Statistics version 25 (IBM Corp). Descriptive analysis and bivariable analysis were performed before multiple linear regression was used to identify predictors of parental barriers.

**Results:**

The overall mean score of parental barriers was 3.51 (SD 0.83), indicating that the average numbers of barriers experienced by parents were more than 3. The multivariable analysis showed that the predictors of parental barriers included monthly household income (adjusted β=–.03, 95% CI –0.05 to –0.02), parents who worked in public sectors (adjusted β=.18, 95% CI 0.06 to 0.29), positive parental attitude on screens (adjusted β=.68, 95% CI 0.58 to 0.79), low parent self-efficacy to influence child’s physical activity (adjusted β=–.32, 95% CI –0.43 to –0.20), and child screen time (adjusted β=.04, 95% CI 0.02 to 0.06).

**Conclusions:**

The strongest predictor of parental barriers to reduce excessive child screen time was the positive parental attitude on screen time which could contribute to their abilities to limit child screen time. Thus, future intervention strategies should aim to foster correct parental attitudes toward screen time activities among young children.

## Introduction

Screen time refers to the total amount of time a person spends passively on any screen-based technology such as smartphone, tablet, video game, computer, television, or any wearable device [1,2]. The World Health Organization recommends that children aged younger than 2 years should have no access to screen time whereas for those aged 2 to 5 years, sedentary screen time should not exceed 1 hour per day, and less is better [1]. In recent years, the increasing prevalence of excessive screen time among children has become a public health issue of global importance [1,2]. Research studies showed a high percentage of children and youth who exceeded the recommended screen time worldwide [3-5]. For example, a study in the United States reported that on average, children aged 8 months to 8 years were exposed up to 4 hours of background television in a day [6]. Likewise, a study in the United Kingdom reported a high proportion of children aged 6 to 36 months were using screen media, and their screen time increased significantly with age [7]. In addition, studies from two developed countries in Asia (ie, Singapore and Japan) also reported that children aged younger than 5 years were exposed to both television and other devices an average of 4 hours per day [8,9]. Locally, two Malaysian surveys found that 27% to 52% of children aged younger than 6 years were exposed to television and other devices (computers, tablets, and smartphones) for more than 2 hours per day [10,11]. Furthermore, about 68% of older Malaysian children (aged 7 to 12 years) spent an average of 3 hours on screen time daily [12]. Even more worrying is the fact that as high as 74% of Malaysian children aged younger than 2 years have been exposed to screen time, in contrast to the World Health Organization recommendation [11].

Multiple studies have highlighted the undesirable effects of excessive screen time on the developmental, psychosocial, and physical health of children. Among these negative effects were visual symptoms after prolonged screen use [13]; speech delay; and unfavorable development of physical, cognitive, and academic abilities of children [14-18]. Moreover, studies also report increased body fat and obesity as a result of increased food consumption and exposure to unhealthy food advertisements during screen viewing [19]. In view of these adverse effects, parents were advised to limit the screen time of their children based on the appropriate duration recommended for the child’s age [1,2].

During childhood and adolescence periods, parents play a crucial role in preventing the development of sedentary behavior [20]. Several studies have demonstrated the role of parental influences on a child’s screen time behavior [21,22]. However, it was also reported that parents could be facing various barriers in the efforts to reduce excessive child screen time [23-26]. Among these barriers were the lack of affordable alternative activities and poor accessibility to them secondary to weather conditions and transportation issues [23,24,26]. Additionally, parental issues such as parental fatigue, time constraints, and a desire to have time away from children to complete personal tasks also hinder the participation of children in physical activities [24,25,27]. Available studies suggested it is vital to identify such barriers experienced by parents for the establishment of effective intervention in promoting healthy behaviors among children [28]. However, a limited number of studies report on factors associated with parental barriers to reduce a child’s screen time in Malaysia. This study aimed to investigate parental barriers toward the reduction of a child’s screen time and relevant predictors from the aspect of sociodemographic, parental, child-related, and environmental factors among parents of children aged younger than 5 years who attended child health clinics in the Petaling District, Selangor, Malaysia.

## Methods

### Study Design and Location

A cross-sectional study was conducted in 9 health clinics located in the Petaling District from April 2019 to June 2020. This study was conducted in health clinics because all government health clinics in Malaysia offer free child health services such as health screening, developmental and nutritional status assessment, immunizations, and dental health services that are accessible for all local citizens [29,30]. Due to this fact, Malaysian parents, especially those of middle and lower income groups, predominantly used these health clinics. Petaling District was selected as the study site was because it is the most populated district in Selangor, Malaysia, with a high number of young children [31]. Petaling District is urbanized with one of the highest median household incomes [32].

### Study Participants

The study population was Malaysian parents of children aged younger than 5 years attending the child health clinics in the Petaling District. Parents who were illiterate or whose children had physical or mental disabilities or chronic diseases such as heart disease and asthma were excluded. These exclusion criteria ensured that we recruited parents with healthy children whose access to screen media was not influenced by their health condition.

### Sample Size and Sampling Method

The sample size was calculated using the correlation coefficient formula based on the inputs from a previous study of a similar topic [33]. Based on a 95% confidence level, 80% power, and 20% nonresponse rate, a minimum sample size of 737 participants were required. To achieve the sample size, stratified sampling with probability proportionate to sample size was adopted. The sample size for the parent-child dyad in each clinic was made to be proportionate to the average number of children attending the 9 child health clinics in the Petaling District in 2017, totaling approximately 7270. We used the attendance record from each clinic as the sampling frame, and each respondent was selected using a systematic sampling method whereby the k interval obtained was 14. The first parent-child dyad (a parent attending the clinic with a child aged younger than 5 years) was selected using a random number generator that gave all respondents an equal chance of being chosen. By repeating the same method in each clinic, a total of 789 participants were recruited for the study.

### Study Instruments

The study employed a validated study instrument adapted from various studies with a good to excellent level of 1-week test-retest reliability (intraclass correlation coefficient 0.64-0.98). The original English version of the questionnaire consisted of 8 sections and was translated into the Malay language by experts from the Editing and Translation Service, Centre for the Advancement of Language Competence, at University Putra Malaysia. See Multimedia Appendix 1 for questionnaire.

#### Sociodemographic Factors

Information regarding age, gender, ethnicity, education level, household income, employment status, and marital status of respondents was collected. The household income in Malaysia is categorized into 3 income groups based on the median monthly household income reported by Department of Statistics Malaysia: namely, the upper 20% (T20), the middle 40% (M40), and the lower 40% (B40) [32]. These income group definitions are not fixed depending on the Malaysia’s gross domestic product.

#### Parental Factors

Parental attitude toward screen time was evaluated based on parent opinions of statements related to screen time benefits. The scores were then categorized [34,35], and parents who scored above 32 were rated as having a positive attitude. Next, parent perceptions were evaluated regarding the influence of screen time on their child’s physical, cognitive, and social well-being [36]. Higher scores represent a greater perception of positive influence of screen time toward the specific aspect of well-being. In addition, the parent’s self-efficacy in influencing the child’s physical activity reflected the confidence level of the parent in situations related to a child’s physical activity. This variable was classified into 3 groups based on the percentile of the score [26].

Last, parenting style was categorized into 4 groups according to the median split in the Baumrind classification of parent involvement and strictness toward their child [37]. Parental restrictive practices on screen time were assessed based on their sedentary-related restrictive practices in which higher scores reflected more restrictive practices [38]. Parental screen time was defined as the average number of hours a parent spent on television, computers, cell phones, and other electronic devices for leisure purposes excluding work or school use during weekdays and weekends.

#### Child-Related Factors

Data on sociodemographic characteristics (age, gender, and numbers of siblings) of the child were obtained from the parents. The child’s screen time was taken as the average time spent by the child on each media device per weekday and weekend [8]. Child care settings were categorized into home care by parents, home care by other than parents, and childcare center [39].

#### Environmental Factors

Environmental factors refer to the conditions of both the household and neighborhood. Questions on the household environment captured information on the number and type of screen devices in the home including those in the child’s bedroom,, and outdoor play equipment at home. Questions on the neighborhood environment asked about the availability of physical activity facilities in the public areas and perceived safety with regard to the likelihood of criminal activities [40].

#### Parental Barriers to Reduce Excessive Child Screen Time

The outcome of this study was assessed based on 6 statements [34]: (1) there is pressure from society to purchase and use media-related equipment, (2) my neighborhood is not safe for my child to play outdoors, (3) poor weather limits my child’s opportunities to go outside, (4) I need a coping tool to meet the demands of a busy day at work or raising multiple children, (5) I need time to do household chores, and (6) my child really enjoys screen time activities. All items were rated on a 5-point Likert scale ranging from strongly disagree to strongly agree. The mean scores of the responses represented an overall score with regard to parental barriers. The maximum score was 6, with higher scores indicated that parents were facing more barriers.

### Data Collection

A validated self-administered questionnaire was distributed to all respondents. Selected parents were approached during their visits to the child health clinics in the Petaling District.

### Statistical Analysis

Data entry and analysis were performed using SPSS Statistics version 25.0 (IBM Corp). Prior to the analysis, data cleaning was done. Skewness, kurtosis, and histogram were part of the normality testing conducted on continuous data. Following that, descriptive analysis was performed for all variables. Categorical data were described in frequency and percentage, whereas continuous data were shown in mean and standard deviation or median and interquartile range depending on normality test results.

In the next step, bivariable analysis was performed using simple linear regression to determine the association between parental barriers in reducing excessive child screen time and all independent variables. Dummy variables were created for all categorical variables prior to bivariable analysis. Based on the findings from simple linear regression, any variables with *P*<.25 were included in the subsequent multiple linear regression analysis to identify the predictors of parental barriers in reducing excessive child screen time [41]. All variables in the model were tested for interaction and multicollinearity to fulfill the assumption for multiple linear regression analysis. Final findings were presented as adjusted β with 95% confidence intervals with the level of significance set at *P*<.05.

### Ethics

Ethical approval was obtained from the medical research and ethics committee of the Ministry of Health Malaysia (NMRR-19-41-45681 [Investigator-Initiated Research]). All participants provided written informed consent. They were notified that their information would be kept confidential and they could withdraw from the study at any time.

## Results

### Sociodemographic Characteristics of Participants

The majority of the 789 participants were women (662/789, 83.9%), Malays (684/789, 86.7%), and married (781/789, 99.0%). The mean age of the respondents was 31.6 (SD 4.81) years, and 70.6% (557/789) had tertiary education. The median income of parents in this study was MYR 5000 (interquartile range 3000); half (403/789, 51.1%) of the parents belonged to the B40 income group based on the categorization of the Malaysia household income groups [32]. Among the 77.7% (613/789) of parents who were employed, the proportion of parents working in the private and public sectors was similar (Table 1).

**Table 1:** Distribution of respondents according to sociodemographic factors (n=789).

Characteristic		Value, n (%)
**Parent age in years**		
	<30	283 (35.9)
	≥30	506 (64.1)
**Gender**		
	Female/mother	662 (83.9)
	Male/father	127 (16.1)
**Ethnicity**		
	Malay	684 (86.7)
	Chinese	31 (3.9)
	Indian	36 (4.6)
	Other	38 (4.8)
**Parent education level**		
	Primary school and below	14 (1.8)
	Secondary school	218 (27.6)
	Preuniversity and higher	557 (70.6)
**Monthly household income (MYR^a^)**		
	<4360 (B40^b^)	403 (51.1)
	4360-9619 (M40^c^)	310 (39.3)
	>9620 (T20^d^)	76 (9.6)
**Employment status**		
	Public sector	218 (27.6)
	Private sector	221 (28.0)
	Self-employed	174 (22.1)
	Unemployed/housewife	176 (22.3)
**Marital status**		
	Married	781 (99.0)
	Divorced/widowed/separated	8 (1.0)

^a^MYR: Malaysian ringgit. 1 MYR = 0.24 US $.

^b^B40: lower 40%, based on the median monthly household income reported by Department of Statistics Malaysia [32].

^c^M40: middle 40%, based on the median monthly household income reported by Department of Statistics Malaysia [32].

^a^T20: upper 20%, based on the median monthly household income reported by Department of Statistics Malaysia [32].

### Parental Barriers to Reduce Excessive Child Screen Time

The mean score of each barrier reported by parents toward the reduction of excessive screen time as outlined in Table 2 was 3.51 (SD 0.83), and a higher mean score represented a more commonly encountered barrier among the parents. Most parents attributed the need to spend time on household chores, unpredictable weather, and lack of a safe neighborhood that restricted outdoor play as barriers for them to limit screen time exposure in their children. Table 2 shows the distribution of the different barriers experienced by parents in the efforts to reduce screen time among children.

**Table 2:** Distribution of parental barriers to reduce excessive child screen time (n=789).

Barrier statement	Strongly disagree, n (%)	Disagree, n (%)	Somewhat agree, n (%)	Agree, n (%)	Strongly agree, n (%)	Mean (SD)
There is pressure from society to purchase and use media-related equipment (such as mobile devices, computers, and DVD players).	112 (14.2)	251 (31.8)	199 (25.2)	171 (21.7)	56 (7.1)	2.76 (1.15)
My neighborhood is not safe for my child to play outside.	75 (9.5)	213 (27.0)	234 (29.7)	197 (25.0)	70 (8.9)	2.97 (1.12)
Unpredictable weather (such as hot, cold, and rain) limits my child’s chances of playing outside.	53 (6.7)	190 (24.1)	256 (32.4)	229 (29.0)	61 (7.7)	3.07 (1.05)
I need a coping tool to meet the demand of a busy day at work or raising multiple children.	57 (7.2)	226 (28.6)	287 (36.4)	175 (22.2)	44 (5.6)	2.90 (1.01)
I need time to do household chores (such as washing and cooking).	48 (6.1)	163 (20.7)	307 (38.9)	221 (28.0)	50 (6.3)	3.08 (0.99)
My child really enjoys screen time activities.	89 (11.3)	225 (28.5)	280 (35.5)	165 (20.9)	30 (3.8)	2.77 (1.02)

### Bivariable Analysis of Parental Barriers to Reduce Excessive Child Screen Time

Table 3 shows all the factors studied that were significantly associated with parental barriers including sociodemographic, parental, child-related, and environmental factors. For sociodemographic factors, parents of Malay (β=–.29, 95% CI –0.56 to –0.01; *P*=.04), Chinese (β=–.49, 95% CI –0.88 to –0.09; *P*=.02), and Indian (β=–.40, 95% CI –0.78 to –0.02; *P*=.04) ethnicity; combined monthly household income (β=–.04, 95% CI –0.06 to –0.02, *P*<.001), and employment status in terms of parents who worked in the public sector (β=.16, 95% CI 0.00 to 0.32, *P*=.045) or were unemployed/housewife (β=.18, 95% CI 0.01 to 0.34, *P*=.04) were among the factors that were significantly associated with parental barriers.

Parental factors found to be positively associated with parental barriers included parental attitude on child screen time (β=.78, 95% CI 0.66 to 0.89; *P*<.001), perception on the influence of screen time on cognitive well-being (β=.04, 95% CI 0.02 to 0.06; *P*=.001) and social well-being (β=.05, 95% CI 0.02 to 0.08; *P*=.001), as well as parental screen time (β=.18, 95% CI 0.06 to 0.31; *P*=.003). On the contrary, a neglectful type of parenting style (β=.15, 95% CI 0.01 to 0.28; *P*=.03) and low self-efficacy to influence child’s physical activity (β=–.43 95% CI –0.58 to –0.28; *P*<.001) were negatively associated with parental barriers.

In addition, 3 of the 5 child-related factors were significantly associated with parental barriers in reducing a child’s screen time. The significant factors included number of children (β=.18, 95% CI 0.06 to 0.30; *P*=.003), child’s age (β=.01, 95% CI 0.00 to 0.01; *P*=.001), and child’s screen time (β=.07, 95% CI 0.05 to 0.09; *P*<.001).

Environmental factors significantly associated with parental barriers included the presence of screen devices in child’s bedroom (β=–.19, 95% CI –0.35 to –0.03; *P*=.02) and parental perceived safety related to crime (β=.27, 95% CI 0.21 to 0.34; *P*<.001).

**Table 3:** Association between sociodemographic, parental, child-related, and environmental factors with parental barriers (n=789).

Variable			β	SE	*P* value	95% CI
**Sociodemographic factors**						
	Age in years		0.01	0.01	.31	–0.01 to 0.01
	**Gender**					
		Female	Reference	—^a^	—	—
		Male	0.05	0.08	.53	–0.11 to 0.21
	**Ethnicity**					
		Malay	–0.29	0.14	.04^b^	–0.56 to –0.01
		Chinese	–0.49	0.20	.02^b^	–0.88 to –0.09
		Indian	–0.40	0.19	.04^b^	–0.78 to –0.02
		Other	Reference	—	—	—
	**Educational level**					
		Primary school	0.02	0.23	.95	–0.44 to 0.47
		Secondary school	Reference	—	—	–0.24 to 0.02
		Preuniversity	–0.11	0.07	.11	–0.06 to –0.02
	Monthly household income (MYR^c^)		–0.04	0	<.001^b^	–0.06 to –0.02
	**Employment status**					
		Private sector	Reference	—	—	—
		Public sector	0.16	0.08	.045^b^	0.00 to 0.32
		Self-employed	0.03	0.08	.76	–0.14 to 0.19
		Unemployed/housewife	0.18	0.08	.04^b^	0.01 to 0.34
	**Marital status**					
		Married	Reference	—	—	—
		Divorced/widowed/separated	–0.01	0.30	.97	–0.59 to 0.57
**Parental factors**						
	**Attitude on screen time**					
		Negative	Reference	—	—	—
		Positive	0.78	0.06	<.001^b^	0.66 to 0.89
	**Parenting style**					
		Authoritative	Reference	—	—	—
		Authoritarian	0.05	0.12	.66	–0.16 to 0.26
		Indulgent	0.01	0.10	.96	–0.18 to 0.19
		Neglectful	0.15	0.07	.03^b^	0.01 to 0.28
	**Perception on the influence of screen time on child’s**					
		Physical well-being	0.03	0.01	.05	0.00 to 0.05
		Cognitive well-being	0.04	0.01	.001^b^	0.02 to 0.06
		Social well-being	0.05	0.02	.001^b^	0.02 to 0.08
	Restrictive practices on screen time		–0.01	0.01	.10	–0.03 to 0.00
	**Self-efficacy to influence the child’s physical activity**					
		High	Reference	—	—	—
		Moderate	–0.00	0.07	.98	–0.14 to 0.13
		Low	–0.43	0.08	<.001^b^	–0.59 to –0.28
	**Parental screen time in hours**					
		≤2	Reference	—	—	—
		>2	0.18	0.06	.003^b^	0.06 to 0.31
**Child-related factors**						
	**Number of children**					
		Single child	Reference	—	—	—
		2 or more children	0.18	0.06	.003^b^	0.06 to 0.30
	Child’s age in months		0.01	0	.001^b^	0.00 to 0.01
	**Child’s gender**					
		Male	Reference	—	—	—
		Female	0.08	0.06	.18	–0.04 to 0.20
	Child’s screen time in hours		0.07	0.01	<.001^b^	0.05 to 0.09
	**Child care setting**					
		Home care by parents	Reference	—	—	—
		Home care by others	–0.05	0.08	.53	–0.20 to 0.10
		Childcare center	0.01	0.07	.87	–0.13 to 0.15
**Environmental factors**						
	**Additional piece of screen device at home**					
		<3 media devices	Reference	—	—	—
		≥3 media devices	0	0.12	.97	–0.23 to 0.24
	**Screen device in child bedroom**					
		Yes	Reference	—	—	—
		No	–0.19	0.08	.02^b^	–0.35 to –0.03
	**Outdoor play equipment at home**					
		Yes	Reference	—	—	—
		No	0.04	0.06	.52	–0.08 to 0.16
	**Availability of public physical activity facilities**					
		Yes	Reference	—	—	—
		No	0.07	0.06	.29	–0.06 to 0.18
	Perceived safety related to crime		0.27	0.03	<.001	0.21 to 0.34

^a^Not applicable.

^b^Significant at *P*<.05.

^c^MYR: Malaysian ringgit. 1 MYR = 0.24 US $.

### Predictors of Parental Barriers to Reduce Excessive Child Screen Time

A total of 16 factors with *P*<.25 in the simple linear regression were included in the preliminary model. One of the factors, parental perception of safety related to crime, was removed from the model due to its interaction with 2 other factors (public sector employment and monthly household income). The remaining 15 variables showed no multicollinearity and interaction. Hence, all assumptions for multiple linear regression were fulfilled. Forward variable selection method was applied in the final model. The model fits reasonably well with adjusted *R*^2^=.26 and *F* test<0.001.

Table 4 shows that among parents of children aged younger than 5 years, those with low monthly household income encountered more barriers in the efforts to reduce their child’s screen time (adjusted β=–.03, 95% CI –0.05 to –0.02). Furthermore, parents who worked in the public sector were more likely to have a higher parental barrier score when compared with those in the private sector (adjusted β=.18, 95% CI 0.06 to –0.29). Moreover, parents with a positive attitude toward child screen time recorded a higher score of parental barriers than those with a negative attitude (adjusted β=.68, 95% CI 0.58 to 0.79). Parents with low self-efficacy to influence their child’s physical activity also displayed a higher parental barrier score as compared with their counterparts (adjusted β=–.32, 95% CI –0.43 to –0.20). Last, parents whose children spent more hours on screen were expected to face more barriers in the efforts to reduce excessive child screen time (adjusted β=.04, 95% CI 0.02 to 0.06).

**Table 4:** Predictors of parental barriers to reduce excessive child screen time (n=789).

Variable		Adjusted β	SE	*t* test	*P* value^a^	95% CI
Monthly household income (MYR^b^)		–0.03	0	–3.83	<.001	–0.05 to –0.02
**Employment status**						
	Private sector	Reference				
	Public sector	0.18	0.06	3.05	.002	0.06 to 0.29
**Attitude on screen time**						
	Negative	Reference				
	Positive	0.68	0.06	12.37	<.001	0.58 to 0.79
**Self-efficacy to influence a child’s physical activity**						
	High	Reference				
	Low	–0.32	0.06	–5.45	<.001	–0.43 to –0.20
Child’s screen time		0.04	0.01	4.40	<.001	0.02 to 0.06

^a^Significant at *P*<.05.

^b^MYR: Malaysian ringgit. 1 MYR = 0.24 US $.

## Discussion

### Principal Findings

The majority of parents who participated in this study were Malay, females, and married. More than half of them were aged older than 30 years. This finding highlighted the fact that mothers frequently dealt with all matters related to a child’s health, including routine clinic appointments [42]. Moreover, a profile of the respondents also reflected most Malaysian adults, who married in their mid to late 20s [43] and would only bear a child after that age. The high numbers of Malay respondents mirrored the attendees of primary health care facilities in Malaysia, who were mostly Malays with less than one-third being Chinese and Indians [44]. Even though most parents in this study attained preuniversity and higher levels of education, the majority of them belonged to the B40 or M40 group. Furthermore, two-thirds of parents were employed, and the number of parents who worked in the private and public sectors was nearly equivalent. The remaining 22% was self-employed. This finding showed that adults living in urban areas such as the Petaling District have access to various employment sectors in Malaysia.

To the best of our knowledge, no previous studies in Malaysia have reported the barriers experienced by parents in limiting screen time among children aged younger than 5 years. From the analysis, most of the parents experienced an average of more than 3 barriers. This differed significantly from the findings of Jarvis and colleagues [45], whereby parents experienced only one type of barrier. The differences could be due to the different types of barriers presented to the respondents in the studies. Jarvis et al [45] explored barriers that were self-reported by parents through open-ended questions, whereas specific barriers were presented in a self-administered questionnaire to parents in this study. Common barriers encountered by parents in this study (ie, time needed for household chores, weather factor, and coping tools to meet the demand of a busy day) were consistent with previous research findings [23,24,27]. We have found it is a common occurrence among current generations of modern parents to provide screen devices to their young ones as an easy way out [27,46].

In this study, the strongest predictor for parental barriers was the parents’ positive attitude on child screen time. A previous study showed that parents’ positive attitude toward screen behavior could contribute to internal conflict and thus reduce their abilities to limit child screen time [47]. A similar finding was noted in this study. When parents displayed supportive behavior toward screen time and perceived screen time to be beneficial to their child or themselves, it became a barrier for them to place the necessary restriction on screen time activity. This is not surprising because parents’ attitudes toward technology use cast a big influence on how they perceive and value screen time at home [48]. Therefore, our study findings suggested that parents with a more positive attitude toward screen behavior could be subconsciously establishing a home environment that facilitated screen-based activities for their children. Subsequently, this might become a routine habit, leading to early overexposure of children to screen time [5,49]. Eventually, parents would face an uphill battle with the worsening level of barriers toward the reduction of excessive screen time in their children [23].

Furthermore, our study reported that parents with low self-efficacy to influence child’s physical activity encountered more barriers to reduce child screen time when compared with parents with high self-efficacy, as highlighted in a previous study [50]. Parents with low confidence to motivate children in engaging in physical activity might also be less likely to initiate screen time reduction in children. In other words, it would not be convincing for the children to follow the restriction if the parents failed to be the role models in supporting healthy behavior among the children [51].

Apart from that, monthly household income was also a predictor of parental barriers in our study. Parents were likely to have an 0.03 increase in the score of the parental barrier for every 1000 MYR decrement in monthly household income. Lower family income could be linked to certain cost issues and, subsequently, restrict access to healthier choices and substitute activities for screen time, thus constituting a common barrier to support healthy behavior [23,25]. In other words, this could explain why lower income families encounter more barriers: they lack the resources to substitute screen-based activities with other activities as compared with higher income families that can afford to provide alternative activities and facilities for their children to substitute screen time [52,53].

Besides family income, parents’ employment status was another determinant of parental barriers toward the support of active lifestyles in children, based on a systematic review [23]. Our findings were similar in which working parents encountered more barriers to reduce excessive screen time. However, the difference in the challenges encountered by parents working in the public and private sectors needs to be explored further. A recent study showed that workers in the Malaysian public sector frequently experienced work-life conflict as they had difficulty in coping with the pressures of integrating their work and personal lives as a result of the long and inconsistent working hours [54]. This could in turn lead to parenting stress that acts as a potential parental barrier in the reduction of screen time exposure in children [45,55].

Last, previous studies have reported that children’s screen times rose substantially when more barriers were reported by parents [26,34]. This was echoed by our results that demonstrated child screen time as a predictor of parental barriers in the reduction of screen time. The potential reason behind this could be the addictive nature of screen behavior that was integrated into every day routine, thus making it extremely difficult to minimize children’s time spent on screen [5,56].

### Limitations and Strengths

To the best of our knowledge, this is among the first quantitative studies in the local setting that tried to determine parental barriers toward the reduction of screen time in children. The data were collected with a self-administered validated questionnaire using the probability sampling method and examined the association between a dependent variable with many factors. However, this study is not without limitations. Potential recall bias could have occurred during data collection as many factors were only evaluated from the perspective of a single respondent. Furthermore, the samples were only recruited among parents attending the child health clinics in one district. Thus, the study findings should be interpreted with caution in view of the limited generalizability to other populations.

### Future Direction

To overcome barriers encountered by parents, it is essential to enhance parental awareness by providing valuable information through a well-designed health education program. These efforts can help parents overcome the challenges and difficulties of screen time restriction. Health education should consist of information on the recommended screen time behavior for young children. Apart from that, the program should also aim to establish the correct parental attitude on the children’s screen time behavior and provide the relevant skills to improve parental self-efficacy in supporting screen time reduction in children. Importantly, the health education program should target families with low income and parents working in the public sector. Future studies should consider intervention research that pinpoints effective strategies for targeted parental barriers.

### Conclusion

In summary, this study identified 5 predictors of parental barriers to reducing excessive screen time among children, the strongest one being the positive parental attitude toward child screen time. The majority of parents with children aged younger than 5 years experienced more than 3 barriers in their efforts to reduce screen time.

## Appendix

Multimedia Appendix 1 Questionnaire.
