# Supplementary material for: Predictors of Parental Barriers to Reduce Excessive Child Screen Time Among Parents of Under-Five Children in Selangor, Malaysia: Cross-sectional Study
Source: J Med Internet Res. 2021 Apr 13;23(4):e25219. doi: 10.2196/25219 (PMC8080141; doi:10.2196/25219)
Supplement: Multimedia Appendix 1 [file jmir_v23i4e25219_app1.docx]

**Section A: Parents Information**

**INSTRUCTION:** Please mark (√) on the box and/or fill in the blanks below.

| D | D | M | M | Y | Y |
| --- | --- | --- | --- | --- | --- |

1. Date of birth:
2. Age (in years): ________________

| Male |  |  | Female |  |
| --- | --- | --- | --- | --- |

1. Sex:

| No formal education |  |
| --- | --- |
| Primary school |  |
| Secondary school |  |
| Diploma / Certificate |  |
| Bachelor’s degree |  |
| Master’s / PHD |  |

1. Highest Education level:
2. Combined household income: RM____________/month.

| Public sector |  | Private sector |  |
| --- | --- | --- | --- |
| Self-employed |  | Unemployed/Housewife |  |

1. Employment status:

| Married |  |
| --- | --- |
| Divorced / Widow / Separated |  |

1. Marriage status:
2. Current number of children: _________________

**Section B: Child Information**

**INSTRUCTION:** Please mark (√) on the box and/or fill in the blanks below.

| D | D | M | M | Y | Y |
| --- | --- | --- | --- | --- | --- |

1. Date of birth:

| Male |  |  | Female |  |
| --- | --- | --- | --- | --- |

1. Sex:

| Malay |  |  | Other Bumiputera |  |
| --- | --- | --- | --- | --- |
| Chinese |  |  | Others |  |
| Indian |  |  |  |  |

1. Ethnicity:

**Section C: Child Care**

**INSTRUCTION:** Please mark (√) on the box.

1. What kind of child care is your child attending?

| Home Care | By parents |  |
| --- | --- | --- |
|  | Other than parents |  |
| Childcare centre | |  |

**Section D: Total Screen Time Usage**

**INSTRUCTION:** Please mark (√) on the box.

1. On **average,** how long does your **child** spend time on watching television (including video, DVD, PlayStation, Wii, Xbox), computers (including laptops, desktops) or other mobile devices (including mobile phones, tablets, Game Boy) **PER DAY**?

| **Per Weekday** | | | | | | | | | | | | | | | |
| --- | --- | --- | --- | --- | --- | --- | --- | --- | --- | --- | --- | --- | --- | --- | --- |
| Television | | | |  | Computer | | | |  | Mobile devices | | | | |  |
|  | Hour |  | Min |  |  | Hour |  | Min |  |  | Hour |  | Min |  |  |

| **Per Weekend** | | | | | | | | | | | | | | | | | |
| --- | --- | --- | --- | --- | --- | --- | --- | --- | --- | --- | --- | --- | --- | --- | --- | --- | --- |
| Television | | | |  | Computer | | | | |  | | Mobile devices | | | | |  |
|  | Hour |  | Min |  |  | Hour |  | Min |  | |  | | Hour |  | Min |  |  |

1. In a typical week over the past 3 months, how much time per day do you **(parent)** spend your leisure time on watching television, computers or other mobile devices per day?

|  |  | None |  |  |  | From 6 to 10 hours |
| --- | --- | --- | --- | --- | --- | --- |
|  |  | Less than 1 hour |  |  |  | From 11 to 14 hours |
|  |  | From 1 to 2 hours |  |  |  | From 15 to 20 hours |
|  |  | From 3 to 5 hours |  |  |  | More than 20 hours |

**Section E: Parental Factor**

1. Attitude towards screen time.

Below are several reasons why your child is involved in screen time activities (television / computer / mobile devices)

**INSTRUCTION:** Please rate your response accordingly.

| Strongly Disagree  1 | Disagree  2 | Somewhat  Agree  3 | Agree  4 | Strongly Agree  5 |
| --- | --- | --- | --- | --- |

| a | It is good for the development of his/her brain (such as learning aids). |  |
| --- | --- | --- |
| b | It is something my child finds very enjoyable. |  |
| c | It gives me the opportunity to get things done on my own (such as house chores, cooking). |  |
| d | It helps me to handle a busy day at work and/or take care of children. |  |
| e | My child needs/wants time to relax |  |
| f | It provides quality time together with family. |  |
| g | It grabs hold of my child’s attention |  |
| h | It teaches my child to get along with others. |  |

1. Parental perception on the influence of screen time on child’s wellbeing.

To my understanding, below are the impacts of screen time on my child.

|  | |  | Negative influence | No influence | Positive influence |
| --- | --- | --- | --- | --- | --- |
| Physical wellbeing | Heart health | |  |  |  |
|  | Muscle and bone health | |  |  |  |
|  | Maintaining a healthy weight | |  |  |  |
|  | Fundamental movement skills | |  |  |  |
| Cognitive wellbeing | Academic achievement | |  |  |  |
|  | Cognitive development | |  |  |  |
|  | Ability to concentrate | |  |  |  |
|  | Language development | |  |  |  |
| Social wellbeing | School readiness | |  |  |  |
|  | Social competence | |  |  |  |
|  | Self-esteem | |  |  |  |

**INSTRUCTION:** Please mark (√) on the box.

1. Barriers

Reducing my child’s screen time activities (television / computer / mobile devices) may be difficult because:

**INSTRUCTION:** Please rate your response accordingly.

| Strongly Disagree  1 | Disagree  2 | Somewhat  Agree  3 | Agree  4 | Strongly Agree  5 |
| --- | --- | --- | --- | --- |

| a | There is pressure from society to purchase and use media-related equipment (such as mobile devices, computer, DVD player). |  |
| --- | --- | --- |
| b | My neighbourhood is not safe for my child to play outside. |  |
| c | Unpredictable weather (such as hot, cold and rain) limits my child's chances of playing outside. |  |
| d | I need a coping tool to meet a demand of a busy day at work or raising multiple children. |  |
| e | I need time to do household chores (such as washing, cooking). |  |
| f | My child really enjoys screen time activities. |  |

1. Self-efficacy

I am very confident that I can influence my child's physical activity in challenging situations such as when:

| Not Confident  1 | A Little Confident  2 | Somewhat Confident  3 | Confident  4 | Very Confident  5 |
| --- | --- | --- | --- | --- |

**INSTRUCTION:** Please rate your response accordingly.

| a | I do not have much time. |  |
| --- | --- | --- |
| b | My child is engaged in screen time activities. |  |
| c | I feel stressed. |  |
| d | My child does not have a friend to play with. |  |
| e | I cannot think of suggestible activities. |  |
| f | My child is not interested. |  |
| g | I cannot afford to participate in the activity. |  |
| h | My child’s preferred activity is expensive. |  |

1. Parenting style

Below are some ways in which you would generally approach parenting your children:

**INSTRUCTION:** Please rate your response accordingly.

| Strongly Disagree  1 | Disagree  2 | Somewhat  Agree  3 | Agree  4 | Strongly Agree  5 |
| --- | --- | --- | --- | --- |

| a | When someone in the family returns or leaves the home, the person will inform other family members. |  |
| --- | --- | --- |
| b | I will encourage my child to try better when s/he gets a low grade. |  |
| c | I will help my child with the assignment that s/he does not understand. |  |
| d | My child can count on me when s/he has a problem. |  |
| e | I find it very easy to talk openly with my child. |  |
| f | I will spend time just talking with my child. |  |
| g | If my child gets a good grade at school, I will give him/her a compliment. |  |
| h | We do things for fun together regularly as a family. |  |
| i | If my child gets a low grade at school, I suggest to help him/her. |  |
| j | I really know what my child does in his/her free time. |  |
| k | I will try to know where my child is after school. |  |
| l | I really know where my child goes at night. |  |
| m | I really know where my child is after school. |  |
| n | I try to know where my child goes at night. |  |
| o | I try to know what my child does during his/her free time. |  |

1. Parental restrictive practices.

**INSTRUCTION:** Please rate your response accordingly.

| Strongly Disagree  1 | Disagree  2 | Somewhat  Agree  3 | Agree  4 | Strongly Agree  5 |
| --- | --- | --- | --- | --- |

| a | I have to be sure that my child does not watch too much television |  |
| --- | --- | --- |
| b | I have to be sure that my child does not spend too much time on the computer as well as internet |  |
| c | I have to be sure that my child does not spend too much time playing games on hand held devices. |  |
| d | I will switch off the TV if I think my child is watching too much. |  |
| e | I restrict how much time my child spends watching TV |  |
| f | I restrict how much time my child spends using the computer and playing games on hand held devices. |  |

**Section F: Household Physical Environment**

**INSTRUCTION:** Please mark (√) on the box.

1. How many television, computers (laptops, desktops) or other mobile devices (mobile phones, tablets, Game Boy) do you have at home?

|  | 0 device | 1 device | 2 devices | 3 devices | 4 devices or more |
| --- | --- | --- | --- | --- | --- |
| Television |  |  |  |  |  |
| Computers |  |  |  |  |  |
| Mobile devices |  |  |  |  |  |

1. Does your child have television (including video, DVD, PlayStation, Wii, Xbox), computers (including laptops, desktops) or other mobile devices (including mobile phones, tablets, Game Boy) in his/her bedroom?

| Yes |  | No |  |
| --- | --- | --- | --- |

1. Do you provide outdoor playthings (balls, bicycles, scooters, etc.) at your home?

| Yes |  | No |  |
| --- | --- | --- | --- |

**Section G: Neighbourhood Environment**

**INSTRUCTION:** Please mark (√) on the box.

1. Does your neighbourhood have public facilities such as parks or playgrounds where your child can play and be physically active?

| Yes |  | No |  |
| --- | --- | --- | --- |

If yes, how far is the playground by walking distance?

|  |  | Minute |
| --- | --- | --- |

1. How much do you agree with the statements below about the crime safety of your neighbourhood environment?

**INSTRUCTION:** Please rate your response accordingly.

| Strongly Disagree  1 | Disagree  2 | Somewhat  Agree  3 | Agree  4 | Strongly Agree  5 |
| --- | --- | --- | --- | --- |

| a | There is a high crime rate in your neighbourhood. |  |
| --- | --- | --- |
| b | The crime rate in your neighbourhood makes it unsafe for your child to walk (alone or with anyone) at night. |  |
| c | I am worried to allow my child to play outside alone in my house area (such as compound, walkway and public area of apartment) because I am afraid that my child will be kidnapped or hurt by a stranger. |  |
| d | I am worried to allow my child to be outside with his/her friends because I am afraid that s/he will be kidnapped or hurt by a stranger. |  |
| e | I am worried to allow my child to play or walk alone or with his friends in the neighbourhood and ordinary streets because I am afraid that my child will be kidnapped or hurt by a stranger. |  |
| f | I am worried to allow my child to be alone or with his/her friends in the local or nearby park because I am afraid that my child will be kidnapped or hurt by a stranger. |  |

1. How much do you agree with the statements related to pedestrian walking safety of your neighbourhood environment?

**INSTRUCTION:** Please rate your response accordingly.

| Strongly Disagree  1 | Disagree  2 | Somewhat  Agree  3 | Agree  4 | Strongly Agree  5 |
| --- | --- | --- | --- | --- |

| a | There is so much traffic along nearby streets that it makes it difficult or unpleasant for my child to walk (alone or with someone) in our neighbourhood. |  |
| --- | --- | --- |
| b | Most drivers drive at high speed in our neighbourhood. |  |
| c | Our neighbourhood streets has good lighting during the night. |  |
| d | There are crosswalks and signals to help walkers cross busy streets in our neighbourhood. |  |
| e | There are plenty of exhaust fumes when walking in our neighbourhood. |  |

**Section H: Child’s Anthropometric Measurement**

**INSTRUCTION:** To be filled by researcher only.

| Weight (kg) |  |
| --- | --- |
| Height (cm) |  |
| BMI z-score |  |

**END OF QUESTIONNAIRE.**

**THANK YOU!**
